# Diagnostic validity of the *STRATIFY* and *Downton* instruments for evaluating the risk of falls by hospitalised acute-care patients: a multicentre longitudinal study

**DOI:** 10.1186/s12913-017-2214-3

**Published:** 2017-04-17

**Authors:** Marta Aranda-Gallardo, Margarita Enriquez de Luna-Rodriguez, Maria J. Vazquez-Blanco, Jose C. Canca-Sanchez, Ana B. Moya-Suarez, Jose M. Morales-Asencio

**Affiliations:** 1 Department of Nursing, Agencia Sanitaria Costa del Sol, Autovia A7 km. 187, 29603 Marbella, Malaga Spain; 2 Faculty of Health Sciences, University of Malaga, Malaga, Spain

**Keywords:** Falls, Adverse events, Clinical safety, Risk assessment, Reliability and validity, STRATIFY, Downton index, Inpatient

## Abstract

**Background:**

Falls are major adverse events in hospitals. The appropriateness of using risk assessment instruments for falls in hospitals has recently been questioned, although the research performed in this respect presents some methodological shortcomings. The purpose of the present study is to evaluate the accuracy of the Downton and STRATIFY instruments to determine the risk of falls and to predict their incidence in acute care hospitals in the public health system in Andalusia (Spain).

**Methods:**

A longitudinal, multicentre prospective study was made of a cohort of patients recruited between May 2014 and March 2016. The risk of falls was assessed using each of the above instruments during the first 24 h after hospital admittance, with later re-evaluations every 72 h until discharge. Descriptive statistics were obtained, bivariate and multivariate analysis were performed. The diagnostic validity of the process was assessed by calculations of sensitivity, specificity, positive and negative predictive values and ratios of positive and negative likelihood. ROC curve analysis was performed for both instruments.

**Results:**

For this study, 1247 patients were recruited, of whom 977 completed all the follow-up assessments. Twenty-three of these patients (2.35%) suffered 24 falls. ROC curve analysis showed that the optimal cut-off point for each assessment instrument was below that described by the authors: AUC STRATIFY = 0.69 (95% CI: 0.57–0.8); AUC Downton = 0.6 (95% CI: 0.48–0.72). With a cut-off point of 1, the sensitivity of STRATIFY was 47.6% and its specificity, 85%. With a cut-off point of 2, Downton presented a sensitivity of 66.7% and a specificity of 55.3%.

**Conclusions:**

The Downton and STRATIFY falls risk assessment instruments presented little utility as means of detecting the risk of falls among a sample of adult patients admitted to acute care hospitals. Fall prevention in hospitals should be based on the study of individual risk factors.

## Background

Falls are a major public health problem worldwide. It is estimated that annually 424,000 fatal falls occur, which makes this the second global cause of death from unintentional injuries. Falls are the predominant cause of injury among the elderly (aged over 65 years). The highest rates of mortality from this cause correspond to those aged over 60 years [1]. In Europe, falls cause 13.3 to 164.5 deaths per 100,000 population aged over 65 years [2], while 20–30% of elderly persons who fall suffer injuries, ranging from mild to severe, such as hip fractures or head lesions. These lesions reduce mobility and independence and increase the risk of premature death. Among hospitalised patients aged over 65 years, the number of falls suffered in the previous year is a significant predictor of functional impairment, with a negative impact on the performance of basic activities of daily life [3].

In addition to these physical consequences, falls have a psychological impact, involving restricted mobility (sometimes encouraged by the family or health workers), fear of recurrence and loss of self-esteem and independence, which can lead to patients’ modifying their lifestyles.

Furthermore, falls are very costly for the health system, provoking hospital costs in the United Kingdom National Health Service [4], for example, of some 15 million pounds a year (about 92,000 pounds a year for an 800-bed hospital).

These adverse events may occur in any area of health care. Fall rates vary according to health practices, the patients’ environment and the measurement method used, and so statistics for the incidence of falls in hospitals worldwide present great variability: 14.9% of a hospital in Switzerland [5], 8.7% in an acute-care hospital in Australia [6], or 1.6% in a Japanese hospital [7]. In Spain in recent years have reported rates of falls in hospitals ranging between 1.8% [8] or 0.6% in a recent study [9]. The aetiology of this event has been the subject of various epidemiological studies. One recent systematic review showed that the major risk factors for falls are disorders of balance and gait, polypharmacy and a history of previous falls. Other risk factors include advanced age, female gender, visual impairment, cognitive impairment and environmental factors [10].

Based on the risk factors identified traditionally, a number of risk assessment tools have been developed, seeking to reduce the occurrence of falls. However, the use of these instruments has been called into question. Some studies have compared the reliability and validity of fall risk assessment tools against clinical judgment, obtaining poor results of both methods as predictors of falls in hospitals [11]. This paper already showed the difficulty of assessing the reliability and validity of the fall scales due to the adoption by the nurses of measures aimed at preventing them that may alter their results. This is known as “treatment paradox”.

Although the most recent clinical practice guidelines regarding fall prevention highlight the ineffectiveness of these instruments for predicting the risk of falls among elderly hospitalised patients [12], it is also true that, according to a systematic review by our research group [13], many of the studies conducted to evaluate these tools have presented methodological shortcomings, related especially to the calculation of the necessary sample size and a lack of awareness of the effect of periodic re-assessments of the patient. The results of our meta-analysis showed that the STRATIFY scale achieved greater diagnostic validity, with a diagnostic OR of 7.64 (95% CI: 4.86–12.01) than the Morse [14] and Hendrich II Fall Risk Model [15] instruments. The STRATIFY tool has been widely studied, in many care settings, including acute care, geriatrics and rehabilitation. A systematic review and meta-analysis of this instrument, used among hospitalised patients, identified 24 references [16], and subsequent publications have validated it for use in hospitals [6, 17].

The concern among health services to reduce the incidence of falls, as a key element in patient safety strategies, has led various countries, including Australia [18] and Canada [19], to recommend the use of fall risk assessment tools. In Spain, the Strategy for Patient Safety in the Andalusian public health system recommends including the assessment of falls risk in the comprehensive assessment of patients carried out in the first 24 h of hospitalisation [20], and the Downton index is specifically endorsed [21]. Paradoxically, the only published study that has evaluated the diagnostic accuracy of this instrument and the time required for its completion, in comparison with other instruments for acute care hospital patients, reported unsatisfactory results in relation to their diagnostic validity, the time needed to complete it or the possibility of administering it in all patients studied [22]. The above-mentioned meta-analysis did not include the Downton index, as insufficient studies in this respect were available [13]. These circumstances call into question the appropriateness of using this instrument in hospital care.

In view of the above considerations, it seems appropriate to analyse the assessment of fall risk by STRATIFY in acute care hospitals. Nevertheless, no studies have been conducted to validate this instrument in Spain, although such an analysis is recommended before their use [23]. Nor have studies been conducted to validate the Downton index in our country. Therefore, we believe a validation study of these instruments is required, one that addresses and overcomes the major limitations observed in previous studies, in order to determine which approach provides the best results. However, the main reason supporting the realization of this study is the search for a response to the discordance between the recommendations of the clinical practice guidelines and those of the health services (specifically in Andalusia) as discussed above regarding the use of fall risk assessment tools in hospitals.

### Aim

The aim of this study was to evaluate the diagnostic accuracy of the STRATIFY and Downton instruments to detect the risk of falls among acute care hospital patients in Andalusia (Spain) and to determine the effect on diagnostic performance of the periodic re-evaluation of patients with these instruments.

## Methods

### Design

A longitudinal, multicentre, prospective cohort study, with follow-up, was performed.

### Sample

To calculate the sample size, the prevalence of falls reported in previous studies was taken into consideration. In this respect, Härlein reported a falls prevalence in hospitals of 5.4% [24]. The sensitivity of each instrument was also calculated, as the parameter of greatest interest in such measures, in view of the potentially fatal consequences of these adverse events. The sensitivity to STRATIFY in hospitals is estimated at 68.2% [22], and that of the Downton index at 92% [25]. Assuming an alpha value of 0.05 and losses to follow up of 15%, the total sample size required to evaluate the diagnostic accuracy of both instruments was 1183 subjects.

### Participants

The study was conducted in five acute care hospitals in Andalusia, Spain.

The study subjects were all adult patients (aged over 16 years) admitted to inpatient units at these hospitals, with an expected stay exceeding 48 h and who agreed to participate in the study, for which purpose their signed informed consent was given. The following subjects were excluded from the study: obstetric, paediatric and psychiatric patients, those treated in A&E departments, medical and surgical day-care units, short-stay patients, patients in areas of post-surgical recovery, any subjects who for whatever reason could not be followed up for the periods determined, and those who refused to participate.

### Data collection

The data were compiled from May 2014 to March 2016. In each hospital, a study coordinator identified the participant units, excluding those which usually treated patients subject to the exclusion criteria established (post-anaesthesia recovery units, A&E Department, obstetrics units, paediatric care, day care, short-stay units and mental health care). The study coordinator informed the research team of the number of hospital beds concerned and their distribution within the participating units. In order to eliminate a possible selection bias for each such unit, the beds were randomised, such that patients admitted consecutively to the randomised beds were eligible to participate in the study if they met the inclusion criteria and agreed to participate. The staff assigning the beds to the patients was blinded to this randomization. Only the nurses who participated in the study knew in which beds patients were eligible to be part of the study. Since the study sought to assess the validity of the STRATIFY and Downton instruments under usual clinical practice conditions, these nurses were not blinded to the results of both tools. When a patient presented cognitive impairment or was disoriented, participation in the study and signed informed consent was requested of family members or caregivers.

The following variables were compiled: age, sex, centre, type of unit (medical, surgical or ICU), falls prevention measures in place, number of falls occurred, level of consciousness during the fall, date and time of the fall, circumstances and consequences of falls, and all the items required for the Downton and STRATIFY tools.

The cut-off points of both scales are defined by their authors: for STRATIFY, a score ≥ 2 indicates a “high risk of falls” [26], and for Downton, scores ≥ 3 indicate a “high risk of falls” [27]. Patients with lower scores are considered “low risk of falls” for calculations of sensitivity, specificity and predictive values.

The version of STRATIFY used had been previously subjected to cross-cultural adaptation and content validation [28]. The Downton scale is habitually used in Spain, and in this study we applied the original version, without the translation error that had been detected in the Spanish version of this instrument [29]. This consisted of erroneous assignment of punctuation in three items of the tool.

The tools were administered to the patients by nurses, previously trained in their use by members of the research team, during the first 24 h of hospital admission. Then, every 72 h until discharge the risk of falls was re-evaluated, with both instruments.

The occurrence of falls was verified by three different sources for each case, to minimise the risk of under-reporting: by asking the patient and/or relative directly, by analysis of the falls record kept by the hospital unit, and by examining the patient’s clinical history, in addition to consulting with the nurse responsible. The definition employed for this event was that proposed by the World Health Organization, which defines a fall as “an event which results in a person coming to rest inadvertently on the ground or floor or other lower level” [1]. In all cases of falls, the nurses collaborating with the project filled in a report form stating the circumstances and consequences for the patients.

### Data analysis

By exploratory analysis, descriptive statistics were obtained of the variables, including measures of central tendency and dispersion, or percentages, depending on the nature of the data. In every case, the normality of the distribution was assessed by the Kolmogorov-Smirnov test. In addition, the presence/absence of skewness and kurtosis was determined and histograms were obtained of the distributions.

Bivariate analysis was performed using the Student t and chi square tests, according to the characteristics of the variables analysed, when the data were normally distributed. Otherwise, the non-parametric Wilcoxon and Mann-Whitney U tests were used. ANOVA was used, when appropriate, to determine quantitative and qualitative relationships, with measures of central robustness in cases of non-homoscedasticity (determined by the Levene test), applying the Welch test and the Brown-Forsythe test [30]. Diagnostic validity was assessed by calculations of sensitivity, specificity, positive and negative predictive values and ratios of positive and negative probability. ROC curves were analysed to determine cut-off points, assuming non-parametric distribution. In addition, the rates of correct classification (performance test) were calculated. Analyses of predictive validity were performed, using the values obtained throughout the follow-up periods, in order to assess fluctuations in the level of risk and its influence on the diagnostic performance of the scales.

Kaplan-Meier analysis was performed to evaluate the longitudinal evolution of falls, and multivariate analysis, by Cox regression, was performed to determine the contribution of the various components of the scales to the fall risk obtained. In addition, the variables used to characterise the patients were taken into consideration as appropriate.

The level of statistical significance was set at *p* < 0.05 and all analyses were performed with SPSS v.22.0 and EPIDAT 4.0.

## Results

**Fig. 1:**
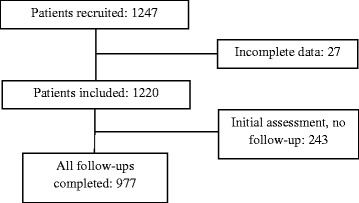
Flow chart of the study

During the study period, 1247 patients were recruited. In consequence, 3386 fall risk assessments were made using the STRATIFY and Downton instruments. 27 patients were lost to follow up, due to incomplete data. 243 patients (19.49% of the original sample) only received the initial assessment on admission, and not the scheduled follow-ups every 72 h until discharge, death or transfer to another unit or centre. The reasons were: discharge before 72 h, death of the patient, transfer to another unit or another hospital, refusal of the patient to continue in the study or impossibility to carry out the follow-up on the part of the nurses who participated in the study. Finally, 977 subjects (78.35%) completed the initial assessment and all scheduled follow-ups (Fig. 1). Although one patient received 23 follow-up assessments, 90% of the patients received just six such assessments. The patients were admitted to the following medical units: internal medicine, palliative care, pneumology, cardiology, nephrology and digestive medicine. The relevant surgical specialties were general surgery, thoracic surgery, urology, traumatology and otolaryngology.

The patients’ characteristics are shown in Table 1. The genders were fairly evenly balanced (53% men). The average age of the sample (*n* = 977) was 65.58 (SD 17.55) years. By type of unit, 59.2% of the patients recruited were treated in specialist medical units, and the average age of these patients (67.53 years, SD 17.18) was higher than that of the surgical patients (62.34 years, SD 18.65). The difference was 5.19 years (95% CI: 2.27–8.12) *p* < 0.001.

**Table 1:** Characteristics of the sample population

	Total sample *N* = 977 *N* (%) or Mean (SD)	Fallers *N* = 23 *N* (%) or Mean (SD)	Non-fallers *N* = 954 *N* (%) or Mean (SD)
Sex			
Male	518 (53)	10 (43.5)	508 (53.3)
Female	459 (47)	13 (56.5)	446 (46.7)
Mean age	65.58 (17.55)	73.57 (14.19)	65.39 (17.58)
Centre			
ASCS	687 (70.3)	14 (60.9)**	673 (70.5)
HUVV	116 (11.9)	4 (17.4)	112 (11.7)
HRUM	78 (8)	3 (13)	75 (7.9)
ASAGA	55 (5.6)	2 (8.7)	53 (5.6)
ASAGM	41 (4.2)	0	41 (4.3)
Type of unit^a^			
Medical	578 (59.2)	18 (78.3)	560 (58.7)
Surgical	334 (34.2)	5 (21.7)	329 (34.6)
ICU	64 (6.6)	0	64 (6.7)
Mean STRATIFY score	0.75 (0.83)	1.50 (1.22)*	0.74 (0.83)
Mean Downton score	2.57 (1.88)	3.33 (2.26)*	2.56 (1.88)

^a^ Lost = 1

*p < 0.001

**p < 0.05

With respect to the primary outcome of the study, 23 patients fell, accumulating a total of 24 falls, with an incidence of 2.35%. All these falls occurred between the first and eighth follow-up assessments, i.e., within a hospital stay of 21 days. The highest concentration of falls (9) occurred around the third day, and 15 falls during the first week. The patients who fell were aged between 33 and 93 years, with a mean age of 73.57 years (SD 14.19). The fallers were significantly older than the non-fallers (*p* = 0.015). Women suffered more falls (*n* = 14) than men (*n* = 10), but this difference was not significant (*p* = 0.565).

The STRATIFY scores, for all the assessments obtained (*n* = 3386) ranged from 0 to 5 points, with a mean score of 0.75 (95% CI: 0.72–0.78). The Downton index scores ranged from 0 to 9 and the mean score was 2.57 (95% CI: 2.50–2.63). According to the cut-off point defined by the STRATIFY authors, this instrument identified a “risk of falls” in 16.2% of cases (*n* = 548), but in fact a fall occurred in only 1.8% of these cases (*n* = 10). With the Downton index, 45.5% of the assessments recorded a “high risk of fall” (*n* = 1541), but the event only occurred in 0.9% of these cases (*n* = 14).

By type of unit, the mean STRATIFY score was higher among surgical patients (mean score 0.82; 95% CI: 0.77–0.87) than in those treated in the ICU (mean score 0.23; 95% CI: 0.17–0.29; *p* < 0.001) and also higher than among the medical patients (mean score 0.77; 95% CI: 0.74–0.81; *p* < 0.001). In contrast, with the Downton index, the medical patients obtained a mean score (2.72; 95% CI: 2.64–2.81) that was, significantly, 0.29 points higher than that of the surgical patients (2.44; 95% CI 2.32–2.55; *p* < 0.001) and 0.55 points higher than that of the ICU patients (1.89; 95% CI; 1.74–2.04; *p* < 0.001).

**Fig. 2:**
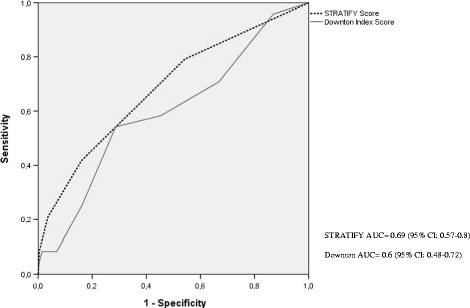
ROC curves for STRATIFY and Downton for the whole study sample

Analyses of sensitivity and specificity, for both instruments, were performed using ROC curves (Fig. 2). STRATIFY obtained a larger area under the curve (AUC) than Downton, and this result was statistically significant. Thus, for STRATIFY, AUC = 0.69 (95% CI: 0.57–0.8; *p* = 0.002) while for Downton, AUC = 0.6 (95% CI: 0.48–0.72; *p* = 0.1). In addition, a subgroup analysis was conducted, of 597 patients aged over 65 years, because previous studies have shown that this subgroup tends to suffer most falls. For STRATIFY, the area under the curve was 0.63 (95% CI: 0.50–0.77; *p* = 0.043) while for the Downton index it was 0.55 (95% CI: 0.40–0.70; *p* = 0.450).

The optimal cut-off point for STRATIFY, in our study, was 1, in contrast to the value of 2 described by the author of the instrument. For the Downton index, too, the optimum cut-off point found was lower than that described by the author of the scale. The sensitivity, specificity, predictive values and likelihood ratios of both instruments at the optimal cut-off points in our study, together with those described by the authors, are shown in Table 2.

**Table 2:** Diagnostic validity by optimal cut-off points, in the present study and as defined by the authors of the instruments

	STRATIFY Cut point = 1	STRATIFY Cut point = 2	DOWNTON Cut point = 2	DOWNTON Cut point = 3
Sensitivity	47.6%	41.0%	66.7%	58.0%
Specificity	85.0%	84.0%	55.3%	54.0%
PPV	10.9%	1.8%	5.50%	0.90%
NPV	97.7%	99.5%	97.7%	99.5%
LR+	3.18	2.6	1.49	1.28
LR-	0.61	0.69	0.60	0.76

*PPV* positive predictive value, *NPV* negative predictive value, *LR* likelihood ratio

The mean scores for each scale, at each follow-up moment, were calculated during the patients’ hospital stay. As noted above, the highest numbers of falls were recorded during the second follow-up moment, and in this assessment the scores, with both STRATIFY and the Downton index, were higher among fallers than non-fallers. For all assessments, this pattern was repeated in the case of STRATIFY, but not with the Downton index.

**Fig. 3:**
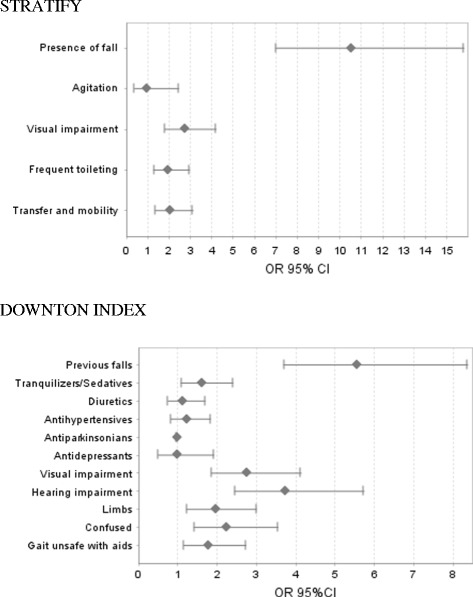
Odds ratio (OR) for the STRATIFY and Downton items in fallers and non-fallers

An analysis was conducted to determine the differential characteristics in the STRATIFY and Downton items between fallers and non-fallers. Significant differences were found in all of the STRATIFY items except the patients’ state of distress; in the Downton index, all items presented significant differences except the consumption of diuretics, antihypertensives, antidepressants and anti-Parkinson medication. In both cases, the highest OR corresponded to the item referring to previous falls: with STRATIFY this item presented an OR = 10.52 (95% CI: 7.00–15.80) *p* < 0.001, and with the Downton index, OR = 5.54 (95% CI: 3.70–8.37) *p* < 0.001. Figure 3 shows the OR for all items for each instrument among fallers and non-fallers, and the corresponding degree of significance.

Finally, the proportional hazard analysis applied to the items of both instruments, to determine which were independent predictors of falls during hospital stay, adjusted for age and sex, showed that “admission after a fall” obtained the highest hazard ratio for falls, followed by “visual impairment”, “frequent toileting” and “previous falls”. In no case did the overall value of the scale achieve discriminatory power (Table 3).

**Table 3:** Cox regression proportional hazard model

	B	HR (95% CI)	*p*
Age	0.01	1.02 (1.00 to 1.03)	0.033
Sex (1: Male 2: Female)	−0.26	0.77 (0.52 to 1.15)	0.208
Admission after a fall	1.77	5.88 (3.24 to 10.67)	0.000
Visual impairment	1.10	3.00 (1.92 to 4.69)	0.000
Frequent toileting	0.75	2.11 (1.25 to 3.57)	0.005
Previous falls	0.70	2.02 (1.16 to 3.50)	0.013
Risk of fall (STRATIFY)	0.16	1.18 (0.65 to 2.13)	0.593
Risk of fall (Downton)	−0.51	0.60 (0.35 to 1.04)	0.067

Dependent variable: fall

## Discussion

The aim of this study was to test and compare the performance of two instruments used to predict the risk of falls in acute care hospitals, and to overcome some deficiencies observed in previous studies in this field. Among its strengths, the present study determined the sample size needed, and in fact this number was exceeded during recruitment. Nevertheless, the occurrence of falls (*n* = 24) was very low. Another strength of this study was its prospective, multicentre nature, with the participation of five hospitals, as well as the systematic, periodic re-assessment of patients every 72 h, producing 3386 assessments. This approach, not adopted in previous studies of this type, enabled us to determine the evolution of the scales during the clinical course of the patients studied.

Referring to the event studied, there were 24 falls, corresponding to 23 patients. The rate of falls obtained (2.35%) is similar to that described in previous studies in Spanish hospitals [8]. The greatest number of falls (*n* = 9) occurred during the first 72 h of hospital stay. Regarding risk factors for falls, it was confirmed that increasing age was associated with the incidence of falls, as reported previously [10]: the average age of the fallers (73.57 years, SD 14.19) was significantly higher than for non-fallers (65.39 years, SD 17.58). What was not confirmed was the hypothesis that female sex was a risk factor for falls, as suggested in a recent systematic review [10]. In our study, although women suffered more falls (*n* = 13) than men (*n* = 10), this difference was not statistically significant (*p* = 0.565). However these results should be interpreted with caution due to the low occurrence of falls.

With respect to risk factors for falls, another systematic review claimed that in hospitals the history of previous falls was strongly related to the occurrence of further falls (OR = 2.85) [31]. This was confirmed in our own study. Thus, the OR for both STRATIFY and Downton showed that the items referring to the history of falls represented the highest likelihood of the occurrence of falls, with respect to any other item (10.52 in STRATIFY and 5.57 in Downton). In this regard, one of the recommendations that could be made to personnel in acute care hospitals is that preventive measures should be maximised for patients with a history of previous falls, especially during the first 72 h following admission.

The mean scores for STRATIFY (0.75, 95% CI: 0.72–0.78) and Downton (2.57, 95% CI: 2.50–2.63) were very low, and lower than the cut-off point reported by the authors of each instrument. The discrimination of a “high risk of falls” differed widely between the two scales: with STRATIFY 16.2% of patients were in this situation, compared to 45.5% identified as such by the Downton index. This difference may arise from the different risk factors investigated by each instrument: while both take into account previous falls and present mobility, there are differences in the assessment of sensory deficits, medication use (not contemplated by STRATIFY), agitation or confusion, and the need of frequent toileting (not assessed by Downton index).

The instruments’ accuracy was also very low with the original cut-off points. With STRATIFY, only 1.8% of the cases identified as “high risk” produced a fall, while only 0.9% of the “high risk” situations identified by the Downton index actually resulted in a fall. These results show that the two instruments have very poor sensitivity values, according to the optimal cut-off value that we identified (STRATIFY: 47.6%; Downton: 66.7%), this being the parameter of greatest interest with respect to preventing the event in question, i.e. the proportion of fallers identified as being at “high risk of falls”. With the cut-off score described by the authors, the results were even poorer (STRATIFY: 41%; Downton: 58%). One possible explanation for these results may be the one already offered by Myers and Nikoletti:*“patients who were assessed to be at high risk for falls were, in fact, at high risk, but because of the fall prevention interventions in place on the study wards these ‘potential’ falls were prevented”* [11]. Failure to implement measures to prevent falls would be an ethical problem in addition to a malpractice of the nurses who care for these patients. This “treatment paradox” is a fact to take into account in this type of studies.

A previous prospective study comparing four instruments aimed at assessing the risk of falls, including STRATIFY and Downton, obtained higher sensitivity values for both scales (68.2% and 81.8% respectively) and lower specificity values (66.4 and 24.7%), although in this case the sample was composed of 135 acute-care inpatients, prior calculation of the sample size was not reported and only an initial assessment was performed, without any subsequent re-assessments [22]. No other prospective validation studies have been published regarding the use of the Downton index in hospitals. On the other hand, several studies of the diagnostic validity of STRATIFY have been conducted. The results obtained by the author of the instrument, for a cut-off point ≥ 2 in its local and remote validation showed better sensitivity values (93 and 92.4% respectively) and similar values of specificity (87.7 and 68.3%) [26] compared to the present study: sensitivity 41% and specificity 84%. These results were reproduced in a recent study: sensitivity 80%, specificity 61.4% [32].

The main differences between them are: the sample size consisting of 217 (local validation), 331 (remote validation) [26] and 217 patients [32] versus 1220 in the present study; a weekly assessment [26] or the abscense of re-assessments [32] compared with the one conducted every 72 h in our study. These studies focused on people over 65 years while the analysis of the subgroup of patients aged over 65 years in the present study did not produce substantial changes in the diagnostic capacity of the assessment instrument. This may reinforce the theory of the “treatment paradox” [11], since in this group of patients, possibly more fragile, protective measures are established in a systematic way. This situation makes impossible to know the real accuracy of the fall risk assessment tools, as already mentioned.

Our analysis of ROC curves corroborated the scant diagnostic validity of STRATIFY and Downton. An assessment instrument is assumed to be reliable when the area under the curve (AUC) is above 0.7 [33]. In our study, this was not the case with the Downton index. Although STRATIFY obtained an AUC = 0.69 (95% CI: 0.57–0.80; *p* = 0.002), close to the reliability frontier, (as mentioned above) it obtained poor results in terms of sensitivity. Therefore, we have little confidence in either of these instruments as a means of determining a diagnostic decision.

Indeed, the very author of STRATIFY has pointed out, in a systematic review and meta-analysis, that this instrument may not be optimal for identifying individuals at high risk of falls [16]. After overcoming the methodological deficiencies observed in earlier studies, our results are in line with the recommendations of the NICE guide [12], in that risk assessment instruments should not be used to identify patients at risk of falls in hospitals. This conclusion may be disheartening for clinicians accustomed to using scales to quantify patient risks. Despite being a directive of some health systems like the Andalusian, nurses will prove that the time invested in completing these instruments has been in vain. The gap between evidence-based recommendations and health systems policies is obvious and we must work to eliminate it. In the case of fall prevention it seems to be well proven that a valid assessment instrument has yet to be developed. Until this occurs, our best approach is to heed the clinical judgment of the nurses involved, a yardstick that has not yet been surpassed by any scale, as noted by Meyer in 2009 [34], and to investigate the risk factors particular to each patient, regarding especially his/her previous history of falls, and by developing prevention programmes to address these multi-causal risks.

This study has certain limitations: its observational design may lead us to infer conclusions that do not have the clear cause-effect relation that is characteristic of experimental study designs, and residual confounding variables may be associated with the results. Furthermore, the low occurrence of falls (*n* = 24) should lead us to interpret these results with caution: despite our recruiting the calculated sample size, the low incidence of falls (2.35%) limits the statistical power of the results obtained. In addition, the events studied may have been under-reported (due to fear that it would have any negative consequences for the professional notifying the event), although we sought to offset this possibility by actively questioning patients and families, by reviewing the notes taken by healthcare workers and by examining the records of falls at each participating centre. Moreover, the effect of the “treatment paradox”, as already mentioned, is another of the limitations of this study, being difficult to avoid.

## Conclusions

The Downton and STRATIFY falls risk assessment instruments have proved to be of scant utility as tools for detecting the risk of falling, among a sample of adult patients admitted to acute care hospitals. Guidelines for patient safety programmes should be aimed at promoting a culture of prevention, taking into account individual risk factors, involving the policymakers within organisations and providing appropriate training for health professionals, patients and caregivers.

## Abbreviations

AUC: Area under the curve
CI: Confidence interval
ICU: Intensive care unit
LH: Likelihood ratio
NPV: Negative predictive value
OR: Diagnostic odds ratio
PPV: Positive predictive value
ROC: Receiver operating characteristic
SD: Standard deviation
STRATIFY: St. Thomas risk assessment tool in falling elderly inpatients
